# The Prevalence of *Demodex* spp. Infestation in Dermatological Patients in Northern Poland

**DOI:** 10.3390/life14091196

**Published:** 2024-09-21

**Authors:** Katarzyna Rychlik, Julia Sternicka, Monika Zabłotna, Roman J. Nowicki, Leszek Bieniaszewski, Dorota Purzycka-Bohdan

**Affiliations:** 1 Department of Dermatology, Venereology and Allergology, Medical University of Gdańsk, University Clinical Centre, 80-214 Gdańsk, Poland; katarzyna.rychlik@gumed.edu.pl (K.R.); julia.sternicka@gumed.edu.pl (J.S.); monika.zablotna@gumed.edu.pl (M.Z.); roman.nowicki@gumed.edu.pl (R.J.N.); 2 Mycology Outpatient Clinic, University Clinical Centre, 80-214 Gdańsk, Poland; 3 Clinical Physiology Unit, Medical Simulation Centre, Medical University of Gdańsk, 80-210 Gdańsk, Poland; leszek.bieniaszewski@gumed.edu.pl

**Keywords:** *Demodex*, parasite, parasitic diseases, skin manifestation, skin diseases

## Abstract

The presence of *Demodex* spp. mites on the skin is a common phenomenon in the human population. In most people, it is an asymptomatic infestation, but in some patients, it can contribute to the occurrence of diseases such as demodicosis, rosacea, or blepharitis, as well as non-specific symptoms. In this study, the results of tests assessing the presence of *Demodex* spp. by direct microscopic examination (DME) in 2508 patients from northern Poland with the suspicion of demodicosis were retrospectively analyzed. A total of 3684 tests were performed. Among them, only 1585 (43.02%) revealed the presence of *Demodex* spp., while 2099 (56.98%) were negative. It was shown that in the analyzed population of patients with clinical suspicion of demodicosis, the degree of confirmation of the presence of *Demodex* spp. positively correlated with the patient’s age (*p* = 0.001) and the mite was mainly found on the edges of eyelids and on the facial skin. Additionally, in men, the presence of *Demodex* was more often confirmed than in women (*p* = 0.004). In conclusion, the proper diagnosis of demodicosis still constitutes an important clinical problem due to the non-specificity of symptoms and the low confirmation of clinical suspicions of infestation by DME, especially in lower age groups.

## 1. Introduction

*Demodex folliculorum* (DF) and *Demodex brevis* (DB) are ectoparasites often found on human skin [1]. They live mainly in the sebaceous glands and hair follicles, where they feed on sebum and epidermis [1]. For this reason, the presence of these mites is mostly detected in areas rich in sebaceous glands, mainly on the face—on the cheeks, chin, nose, and on the eyelids, inhabiting eyelash follicles [1,2,3]. Additionally, *Demodex brevis* may occur outside these areas, spreading to the skin of the entire body [2,3]. The prevalence of *Demodex* spp. in the population varies depending on age—the highest density of *Demodex* mites is observed among elderly people, and the lowest in children [2,3,4,5,6]. It is estimated that *Demodex* spp. may occur in up to 100% of the adult population [2,4,7]. In most cases, it is an asymptomatic infestation, but an increased density of these parasites may lead to the development of various skin disorders, especially demodicosis [1,2,3,4,5,6,7,8,9,10]. Some of the most common diseases associated with *Demodex* infestation are folliculitis, rosacea, and ophthalmological complications such as dry eye syndrome, blepharitis, or chalazion [1,2,3,4,5,6,8]. It is believed that this may be related to the colonization of the mites’ bodies by numerous microorganisms, as well as the induction of an inflammatory reaction by the dead bodies of *Demodex* mites within the hair follicles [2,8]. The diagnosis of demodicosis is made by correlating clinical symptoms with the results of direct microscopic examination (DME) or standardized skin surface biopsy (SSSB) [2,5,11,12]. These methods differ in the way they obtain the material, but in both cases, acquired samples are assessed under a light microscope [11]. DME does not visualize hair follicles, which is important for detecting *Demodex* spp. [12]. SSSB, however, has limitations related to the small surface and reduced depth of the collected sample, as well as poor adherence of mites to the microscope slide, potentially resulting in false-negative outcomes [11,13]. DME is considered a simple, effective, and time-efficient method for mite detection; it detects both *Demodex folliculorum* and *Demodex brevis*, while SSSB only identifies *Demodex brevis* [11,14]. The confirmation of demodicosis through DME requires a density of mites above 5 D/cm^2^ [2]. Once the diagnosis is established, treatment is implemented to reduce the amount of *Demodex* spp. on the patient’s skin and to eliminate clinical symptoms. The most commonly used topical drugs are metronidazole, permethrin, benzoyl benzoate, and ivermectin 1% [2,3].

The aim of this study was to determine the prevalence of *Demodex* spp. infestation in a population of patients in northern Poland with clinical suspicion of demodicosis, along with an analysis of the relationship between *Demodex* infestation and sociodemographic factors, based on the results of tests conducted in the Mycological Laboratory of the University Clinical Centre in Gdańsk. So far, there is no study concerning *Demodex* spp. infestation on such a large population in the Pomeranian region of Poland.

## 2. Materials and Methods

The histories of patients diagnosed in the Mycology Outpatient Clinic of the University Clinical Centre in Gdańsk, obtained from paper patient records, were retrospectively analyzed. The study included patients with at least one visit to the Mycology Outpatient Clinic of the University Clinical Center in Gdańsk in the years 2019–2024, referred by dermatologists with clinical suspicion of demodicosis. All patients were exclusively of Polish descent (the population of northern Poland). The inclusion criteria were met by 2508 people. People suspected of *Demodex* spp. infestation were tested in specific locations, depending on the symptoms presented. A direct microscopic examination was conducted using an MB-100 microscope (OPTA-TECH, Warsaw, Poland) equipped with a camera. After clinical evaluation, six eyelashes from both the upper and lower eyelids and/or six eyebrow hairs were collected from patients using tweezers. Skin scrapings from the face were obtained using a sterile scalpel. Hair samples were collected in the amount of 20 strands using tweezers. The obtained material was placed on the microscope slide with a mixture of dimethyl sulfoxide and 20% potassium hydroxide. Then, each sample was evaluated under the microscope at 100× magnification by qualified personnel, who looked for adult specimens and larvae to confirm infestation. A criterion for a positive result was a mite density above 5 D/cm^2^. Each visit of a qualified patient was analyzed in terms of the following information: the gender and age of the patient, the place of residence of the patient (urban/rural), the location of lesions (facial skin/eyelids edges/eyebrows/hair), and the result of direct examination in a light microscope. The obtained data were subjected to statistical analysis using Statistica 13.3 (StatSoft Polska Sp. z o.o. Inc., 2017, Cracow, Poland) software. An analysis of qualitative features was conducted with the χ^2^ test in the Pearson method. Independent variables fulfilling the assumptions for parametric tests were analyzed with the Student’s *t* test. Independent variables that did not meet the parametric test assumptions were analyzed with non-parametric tests (ANOVA equivalents): the U Mann–Whitney test (the comparison of two tests) or the Kruskal–Wallis test (the comparison of many samples). In all tests, *p*  <  0.05 was considered a significant level of statistical significance.

## 3. Results

**Figure 1:**
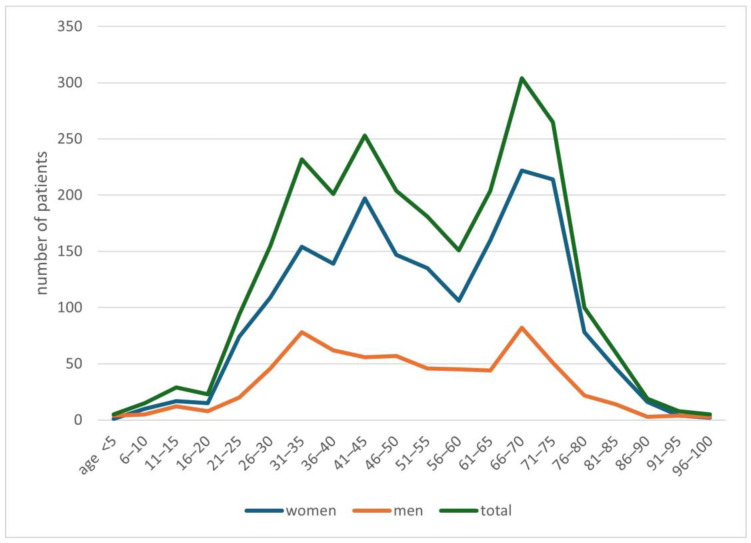
Characteristics of the study population.

In the years 2019–2024, 2508 people with a suspicion of demodicosis were admitted to the Mycology Outpatient Clinic of the University Clinical Centre in Gdańsk. Of this group, 1846 (74%) people were women, while 662 (26%) were men. The average age of patients was 51.9 years, with an average of 52.6 years for women, and for men 50.0 years. Detailed data on the age of patients are presented in Figure 1.

Moreover, 2212 (88.2%) of people suspected of *Demodex* spp. infestation lived in the city, and 296 (11.8%) people in the countryside. Both urban and rural women were statistically more likely to be tested for *Demodex* infestation (*p* < 0.0001). A total of 3684 tests were performed. Among them, 1585 (43.02%) showed the presence of *Demodex* spp., while 2099 (56.98%) had a negative result. It was shown that there is a statistically significant higher probability that at least one of the tests performed during the visit will have a result confirming the presence of *Demodex* spp. if the patient is male (*p* = 0.004) (Table 1).

**Figure 2:**
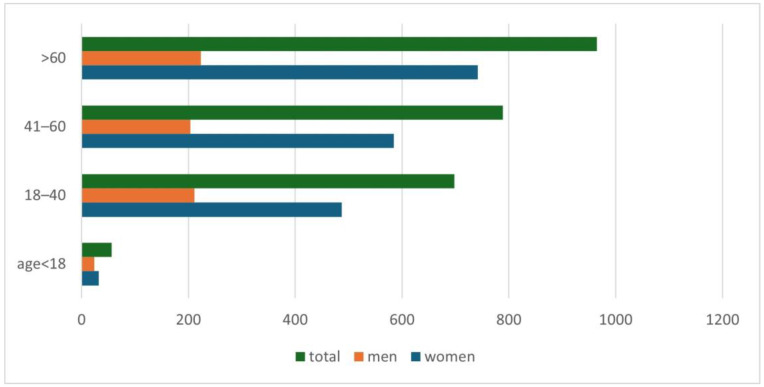
The division of patients with a positive test result for *Demodex* spp., taking into account patients’ age and gender.

**Figure 3:**
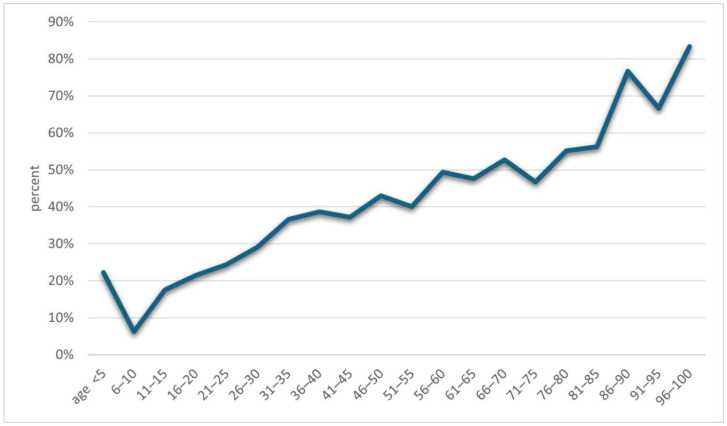
The percentages of results confirming the presence of *Demodex* depending on the patients’ age.

An increase in positive results for *Demodex* spp. was observed along with the age of the examined patients (*p* = 0.001) (Figure 2). A detailed list of results in each age group is provided in Table 2. It was shown that in the analyzed population of patients with clinical suspicion of demodicosis, the degree of confirmation of the presence of *Demodex* spp. using DME positively correlated with the patient’s age (*p* = 0.0001) (Figure 3).

The most frequently assessed area was the skin of the face (*p* = 0.00001) (Table 3). However, locations in which DME showed the presence of *Demodex* mites most commonly were the edges of eyelids, then the facial skin, eyebrows, and hair (Table 3). When analyzing the relationship between the most commonly assessed location of demodicosis lesions and the place of residence in patients by gender, no statistical significance of the obtained results was demonstrated (*p* = 0.18 for women and *p* = 0.95 for men).

**Figure 4:**
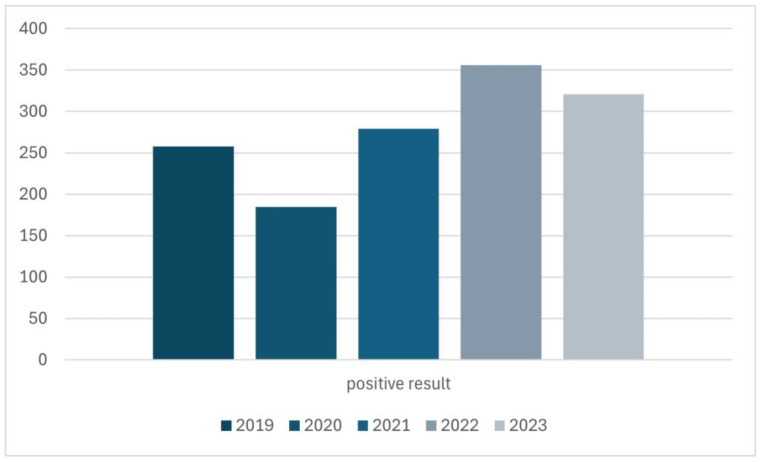
The distribution of positive results for *Demodex* spp. infestation in the years 2019–2023.

The quantitative distribution of results over the years was also investigated (Figure 4). The year 2024 was excluded from the assessment in Figure 4 because only visits from 6 months of this year were analyzed, which would make it impossible to reliably evaluate these data.

## 4. Discussion

The presence of *Demodex* infestation is very common, especially in the adult population. Previous studies in Poland have analyzed the prevalence of this mite in small groups of people, showing its presence in over 40% of respondents [15,16,17]. This study confirms the above data, with the prevalence out of the analyzed group being 46.57% (Table 2). However, it should be noted that this study was conducted with patients whose clinical symptoms prompted specialists to refer them for diagnostic tests. Therefore, these results should not be treated as an adequate illustration of the prevalence of *Demodex* infestation in the general Polish population, but only in the population of people showing symptoms that may suggest excessive proliferation of mites in their skin, so a dermatologist’s patients. The presented statistics also expose that despite the fact that the decision to conduct the test was made by a dermatologist, less than half tests confirmed the presence of *Demodex* spp. in cases of clinical suspicion of infestation, and this rate was particularly low in younger age groups, which well reflects the problem of *Demodex* spp. invasion. Although it is a common problem, the skin lesions caused by this invasion are so non-specific that they can often be confused with other dermatoses, even by experienced dermatologists [18,19]. Another reason for the aforementioned relationship is the fact that the patients included in this study were examined using the DME method. It is not certain whether this type of testing for the presence of *Demodex* spp. is the most effective. In a publication by Ü. Aşkın and D. Seçkin, the higher sensitivity of another method of testing—standardized skin surface biopsy (SSSB)—was demonstrated [12]. Conversely, Chul Hyun Yun et al. reached a conclusion in their research that the DME method exhibited greater value than the SSSB technique [11]. In addition, there are also other, more modern methods of detecting *Demodex* spp. They include reflectance confocal microscopy (RCM) and high-definition optical coherence tomography (HD-OCT). These techniques offer rapid, visual results but have limited application due to low availability and high costs [20]. Despite the lack of consensus on the best method for testing for *Demodex* spp., it may be worthwhile to re-evaluate the patient using a different detection method if there is strong clinical evidence of demodicosis in the patient.

Another issue that has already appeared in previous publications and which is confirmed by this study is the positive correlation of the occurrence of *Demodex* spp. with age—the higher the age of the patient, the higher the probability of being infected with this mite [2,3,4,5,6,15,16,17]. During the analysis, it was shown that the age group in which patients are most at risk for the presence of *Demodex* spp. is people over 60 years of age (Figure 2), among whom 58.65% received a positive test result (Table 2). In turn, in the group of minors, i.e., under 18 years of age, the occurrence of infestation was confirmed in only 14.29% of people. The correlation between age and the frequency of *Demodex* spp. presence confirmation in a given patient is shown in Figure 3. As can be seen, this relationship is especially true for age groups above 15 years of age. This may be related to the small number of patients below this age, which makes it difficult to statistically adequately represent a given population.

According to current knowledge, there is no relationship between gender and excessive proliferation of ectoparasites [9,15,16,17]. The study showed that this is not necessarily true for the patient population in northern Poland and also enabled the observation of a different correlation. Women were statistically more often referred to testing for demodicosis than men. This association had no connection with place of residence—it was true for both urban and rural residents. Despite the fact that women were more likely to be examined, the group in which statistically at least one test for demodicosis turned out to be positive was men (Table 1). The nature of this scientific study did not allow for determining the cause of this difference in results between the sexes. We propose a hypothesis considering differences in preparation for the test—women are more likely to use cosmetic products, such as creams or make-up, which can cause false negative results for *Demodex* spp. [21]. In addition, women have a greater cultural emphasis on meeting certain appearance requirements than men, so minor skin changes can prompt them to visit a dermatologist, which in turn may lead to an increased likelihood of testing for *Demodex* spp. despite less pronounced symptoms [22]. These may be some of the factors conditioning the observed trend.

The prevalence of *Demodex* spp. varies in terms of location. In the population of dermatological patients in northern Poland, the presence of this mite was most often confirmed in the hair follicles of the eyelashes, i.e., on the edges of the eyelids, then on the facial skin, eyebrows, and hair. This coincides with the results of other studies, which have shown an increased occurrence of *Demodex* spp. in areas with increased sebum production, mainly on the facial skin and around the eyelashes [1,2].

This study did not assess the incidence of specific dermatoses depending on the absence or presence of *Demodex*. However, it should be noted that this infestation is a significant clinical problem because it can lead to various skin symptoms. The most common is folliculitis, which is considered to be the initial symptom of increased multiplication of mites on the patient’s skin. This may be accompanied by a burning sensation, itching, and facial erythema [2,4]. The clinical picture of demodicosis may also resemble perioral inflammation, seborrheic dermatitis, and rosacea [2,3,23,24,25]. In turn, research conducted by Zhao YE, et al. proved that *Demodex* spp. infestation is one of the risk factors for the development of acne vulgaris [26]. This was confirmed inter alia by Akçınar UG et al. in. “*Demodex* spp. as a possible aetiopathogenic factor of acne and relation with acne severity and type” [27]. In 2023, another analysis was published showing an increased incidence of *Demodex* spp. in people with blepharitis and chalazion [28]. Different publications have shown that blepharitis, which is associated with *Demodex* spp. infestation, has a strongly negative impact on the well-being of patients suffering from it [29]. This highlights how important the clinical problem of demodicosis is, and that its correct diagnosis and treatment are necessary to maintain a high quality of life for patients.

## 5. Conclusions

In conclusion, the proper diagnosis of demodicosis still constitutes an important clinical problem due to the non-specificity of symptoms and the low confirmation of clinical suspicions of the presence of *Demodex* spp. in direct microscopic examination, especially in lower age groups. In a population of northern Poland patients, *Demodex* spp. most often occurs on the edges of eyelids and on the facial skin but is much less common on eyebrows and hair. Mite infestation shows a positive correlation with the age of patients and, in the Pomeranian region, with the patient’s sex being male. Effective treatment leading to the disappearance of disease symptoms can improve the quality of life of patients, which should be the goal of every diagnostic and therapeutic path.

## Figures and Tables

**Table 1:** The frequency of confirmation of the presence of *Demodex* spp. in a given patient.

	Total Number of Examined Patients	Patients with All Tests Negative	Patients with at Least One Test Positive	Total Number of Tests	Tests Confirming the Presence of *Demodex* spp.
Total	2508	1340	1168	3684	1585 (43.02%)
Women	1846	1018 (55.15%)	828 (44.85%)	2797	1151 (41.15%)
Men	662	322 (48.64%)	340 (51.36%)	887	434 (48.93%)

**Table 2:** The frequency of confirmation of the presence of *Demodex* spp. in given age groups of patients.

			Age (Years)		
	<18	18–40	41–60	>60	Total
Patients	56	698	789	965	2508
Patients with all negative test results (% in a given age group)	48 (85.71%)	454 (65.04%)	439 (55.64%)	399 (41.35%)	1340 (53.43%)
Patients with at least one test positive (% in a given age group)	8 (14.29%)	244 (34.96%)	350 (44.36%)	566 (58.65%)	1168 (46.57%)

**Table 3:** Division of tests for *Demodex* spp. in terms of location.

Location					
Result of Direct Examination	Facial Skin	Edges of Eyelids	Eyebrows	Hair	All
Total	1710	1598	343	33	3684
Positive	580 (36.59%)	889 (56.10%)	108 (6.81%)	8 (0.50%)	1585
Negative	1130 (53.83%)	709 (33.78%)	235 (11.20%)	25 (1.19%)	2099
Percentage of tests confirming the presence of *Demodex* spp. in particular locations	33.92%	55.63%	31.49%	24.24%	

## Data Availability

Data are contained within the article.
